# The Association Between Renal Function Decline and the Incidence of Urothelial Carcinoma: A 16-year Retrospective Cohort Study in Taiwan

**DOI:** 10.1016/j.euros.2021.02.004

**Published:** 2021-03-11

**Authors:** Yung-Hsin Chuang, I-Feng Lin, Xiang Qian Lao, Changqing Lin, Ta-Chien Chan

**Affiliations:** a Institute of Public Health, School of Medicine, National Yang-Ming University, Taipei, Taiwan; b Research Center for Humanities and Social Sciences, Academia Sinica, Taipei, Taiwan; c Jockey Club School of Public Health and Primary Care, The Chinese University of Hong Kong, Prince of Wales Hospital, Hong Kong SAR, China; d Shenzhen Research Institute of the Chinese University of Hong Kong, Shenzhen, China; e Division of Environment and Sustainability, The Hong Kong University of Science and Technology, Hong Kong SAR, China

**Keywords:** Estimated glomerular filtration rate, Fine particulate matter (PM_2.5_), Reduced renal function, Urothelial carcinoma

## Abstract

**Background:**

The incidence of cancer is higher among patients with end-stage renal disease but it remains uncertain whether a mild decrease in renal function affects cancer.

**Objective:**

To measure the effect of impaired renal function, represented by the estimated glomerular filtration rate (eGFR), personal health behaviors, and long-term exposure to fine particulate matter (PM_2.5_) on the risk of urothelial carcinoma (UC) incidence.

**Design, setting, and participants:**

We performed a population-based cohort study of 372 008 participants aged ≥30 yr with no prior cancer history using the MJ health examination database (2000–2015) and UC diagnosis data from the Taiwan Cancer Registry database.

**Outcome measurements and statistical analysis:**

Cox proportional hazards models were used to quantify the association between eGFR and UC incidence.

**Results and limitations:**

We detected 383 UC cases during a median follow-up of 10.3 yr. Low eGFR was significantly associated with UC (*p* value for trend <0.01): compared to eGFR ≥90 ml/min/1.73 m^2^, the adjusted hazard ratio (HR) was 1.36 (95% confidence interval [CI] 0.98–1.88), 1.86 (95% CI 1.22–2.84), and 1.95 (95% CI 1.06–3.56) for eGFR strata of 60–89, 45–59, and <45 ml/min/1.73 m^2^, respectively. The risk remained elevated after stratifying the follow-up duration to check for reverse causality, and the dose-response relationship was stronger for women than for men. Current smoking (HR 1.34, 95% CI 1.02–1.77) and long-term exposure to PM_2.5_ concentrations ≥25.1 μg/m^3^ (HR 1.54, 95% CI 1.14–2.09) both significantly increased the risk of UC incidence. A significant dose-response relationship between PM_2.5_ and UC was also noted (*p*_trend_ < 0.01). Limitations include the retrospective design and limited information on medical history.

**Conclusions:**

Lower renal function showed a dose-response relationship in elevating UC risk. Long-term exposure to PM_2.5_ is also a possible UC risk factor.

**Patient summary:**

People with kidney function that is lower than normal should monitor the health of their kidneys and other organs in the urinary system. Our study confirmed that as well as smoking, exposure to fine particulate matter in the air may be a risk factor for cancers of the urinary system.

## Take Home Message

We found a dose-response relationship between impaired renal function and incident urothelial carcinoma (UC) that was stronger for women than for men. In addition, the study confirms that smoking significantly increases UC risk and reveals that exposure to fine particulate matter (PM_2.5_) may be an emerging risk factor for UC.

## 1 Introduction

The association of chronic kidney disease (CKD)—a well-known public health burden with its high risk of progression to end-stage renal disease (ESRD), high medical expenses, high morbidity, and poor mortality prognosis [1], [2]—with cancer has recently been discussed [3], [4], [5], [6]. Deteriorating renal function leads to retention of metabolic waste products. These uremic toxins can increase DNA damage [7] and induce a state of oxidative stress. Chronic oxidative stress contributes to immune dysfunction and systemic inflammation, both of which play a role in cancer development [8], [9]. It has been well established that cancer incidence increases after renal replacement therapy in patients with ESRD. However, only a few recent studies have investigated whether renal function is associated with cancer risk among patients with early-stage CKD and healthy individuals [4], [5], [6]. The results are also less robust for site-specific cancers except for urinary tract cancer. Therefore, the primary aim of this study was to elucidate the association between renal function and urothelial carcinoma (UC).

In order to measure the real size of an effect, it is important to control all possible confounding factors. Recent studies found that long-term exposure to fine particulate matter (diameter <2.5 μm, denoted PM_2.5_) can increase the risk of CKD [10], [11] and a plausible positive correlation between PM_2.5_ and UC was identified in previous studies [12], [13]. We therefore believe that PM_2.5_ should be considered as a confounder when exploring the association between renal function and UC. We added PM_2.5_ to the statistical model for adjustment to investigate the relationship between PM_2.5_ and UC as a secondary study aim.

We hypothesized that lower renal function elevates UC risk, and that long-term exposure to PM_2.5_ might also be associated with the development of UC.

## 2 Patients and methods

### 2.1 Study population and design

From 2000 to 2015 (16-yr study period), 471 669 people participated in the MJ health examination program [14], yielding 1 093 479 visits in total. At each visit, information on lifestyle behavior was gathered using a standard self-administered questionnaire, and anthropometric measurements and biological test data were collected during the health examination. Each participant provided written consent authorizing use of the data.

Cancer and death records for those participants were obtained by linking the MJ database with the Taiwan Cancer Registry (TCR) and Cause of Death (COD) data sets through encrypted personal identification (ID) by trained staff members from the Health and Welfare Data Science Center, Ministry of Health and Welfare (MOWH), and from the MJ Health Research Foundation. Neoplasms were coded using the *International Classification of Diseases for Oncology, Third Edition* (ICD-O-3) in the TCR (http://tcr.cph.ntu.edu.tw/main.php?Page=N1). In the COD data set, COD was coded using ICD-9 before 2008 and ICD-10 starting from 2008.

This retrospective cohort study used data for individuals who underwent at least one health examination between 2000 and 2015. The date of the first eligible renal function measurement for each participant was set as their cohort entry date (baseline). The follow-up period was defined from baseline to the time of UC diagnosis. Those who did not develop incident UC were censored at the end of the follow-up period (December 31, 2015) or death, whichever occurred first.

**Fig. 1:**
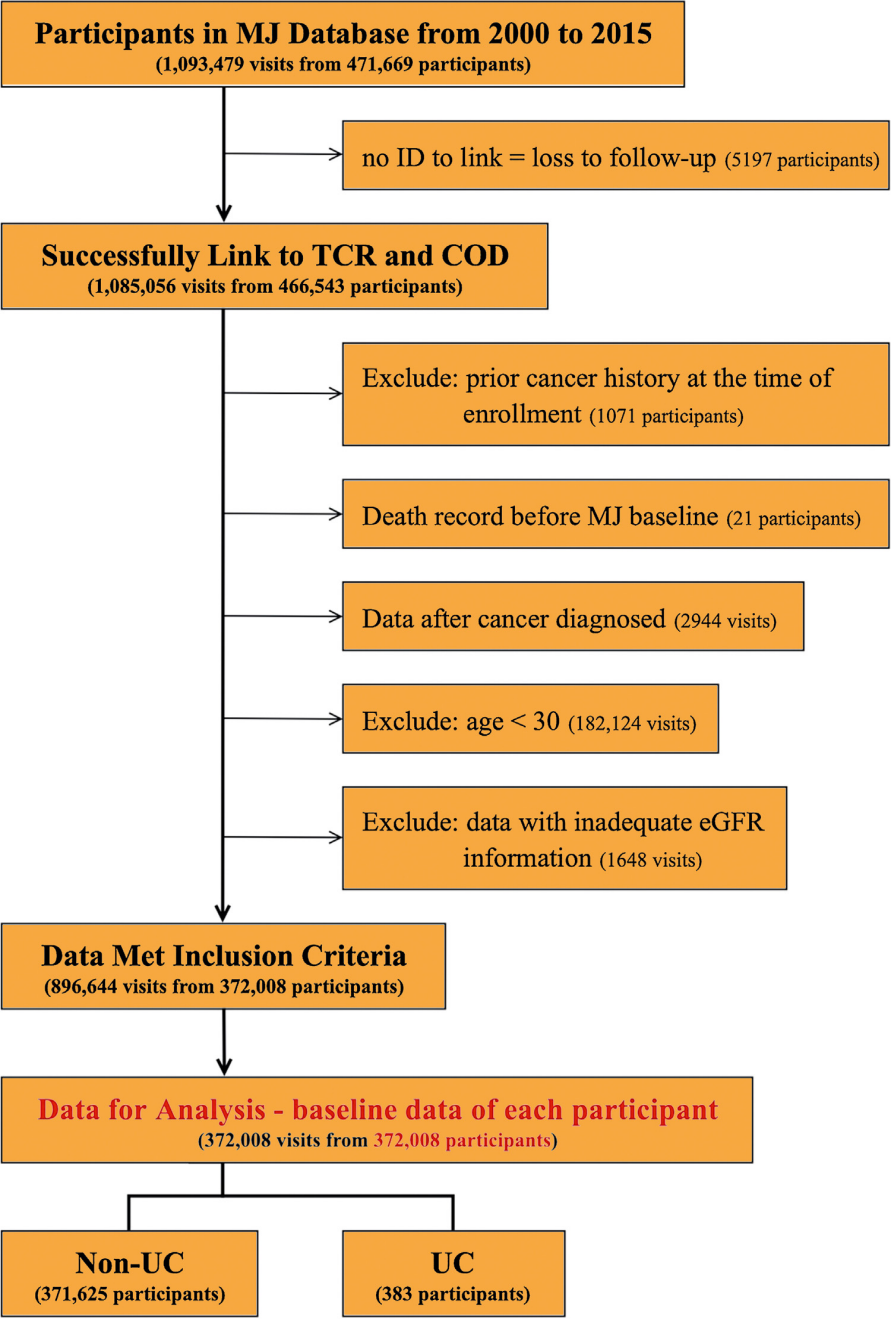
Flowchart of patient inclusion for data analysis. TCR = Taiwan Cancer Registry; COD = cause of death; eGFR = estimated glomerular filtration rate; UC = urothelial carcinoma.

Figure 1 shows details of the participant selection process. Participants with no proper ID number for which a link could be established (*n* = 5197), implying they gave the incorrect ID or were a foreigner, and those who illogically had a death date before their health examination date (*n* = 21) were excluded. Other exclusion criteria were any prior cancer history at the time of enrollment (*n* = 1071), age <30 yr, or missing or inadequate renal function information (estimated glomerular filtration rate [eGFR] ≥200 or <2 ml/min/1.73 m^2^, as these values suggested the measurements were probably incorrect because of technical errors) [15]. The remaining participants (*n* = 372 008) were included in the subsequent analyses. The study was approved by the Academia Sinica review board for biomedical science research (AS-IRB-BM-17044).

### 2.2 Assessment of variables

The primary predictor was renal function at baseline, represented by eGFR calculated using the Chronic Kidney Disease Epidemiology Collaboration equation [15]. We classified eGFR into four categories: ≥90, 60–89, 45–59, and <45 ml/min/1.73 m^2^. Normal eGFR for adults is ≥90 ml/min/1.73 m^2^, so this served as the reference.

The primary outcome of interest was the first incident UC after baseline. UC was defined as carcinoma of the epithelial lining of the urinary tract and can involve the renal pelvis, ureter, bladder, and urethra (ICD-O-3 codes C65.9, C66.9, C67.0-67.6, C67.8-67.9, and C68; Supplementary Table 1). According to location, UC in the renal pelvis or ureter is called upper tract UC (UTUC) and UC in the bladder or urethra is called lower tract UC (LTUC).

To adjust for potential confounders, the following covariates were included in the statistical models. PM_2.5_ exposure at each participant’s address reported in the questionnaire was estimated using a satellite-based spatiotemporal model [16] with high spatial resolution of 1 km × 1 km on the basis of National Aeronautics and Space Administration aerosol optical thickness data [17]. We calculated a 2-yr average PM_2.5_ concentration as the mean PM_2.5_ concentration for the calendar year of the health examination and that for the previous year as an indicator of long-term exposure to ambient PM_2.5_ air pollution. This 2-yr mean PM_2.5_ concentration (in μg/m^3^) was then categorized into quartiles: <18.25, 18.25–22.34, 22.35–25.0, and ≥25.1 μg/m^3^.

All other covariates were defined using baseline health examination data. Demographic data on age, gender, education level, lifestyle behaviors at the time of the study including smoking and drinking habits, and long-term medication were collected via the questionnaire. The presence of proteinuria and hematuria was identified from urine test results. Information on comorbidities was evaluated on the basis of the questionnaire and abnormal health examination records. Diabetes mellitus (DM) was defined as either a reported history of DM diagnosis or current use of antihyperglycemic drugs indicated on the questionnaire or a record of fasting plasma glucose (FPG) > 126 mg/dl during the health examination. Hypertension was defined as a reported history of hypertension diagnosis or current use of antihypertensive drugs. Cardiovascular disease (CVD) was defined as a reported history of CVD diagnosis or current use of cardiac drugs. Nephrosis was defined as a reported history of nephrosis diagnosis. Metabolic syndrome was defined according to the Taiwan Ministry of Health and Welfare criteria (https://www.hpa.gov.tw/Pages/List.aspx?nodeid=221), which requires three or more of the following five measures: (1) abdominal obesity; (2) elevated blood pressure (because we did not obtain actual blood pressure information from the MJ database, reported current use of antihypertensive drugs served as a proxy variable); (3) elevated FPG; (4) elevated triglycerides; and (5) low high-density lipoprotein cholesterol.

### 2.3 Statistical analysis

All analyses were performed using SAS version 9.4 (SAS Institute, Cary, NC, USA). Baseline characteristics for the participants are presented as descriptive statistics. Differences in baseline characteristics across eGFR categories were assessed using one-way analysis of variance or the Kruskal-Wallis test for continuous variables and Pearson’s χ^2^ test for categorical variables, as appropriate.

The hazard ratio (HR) and 95% confidence interval (CI) for the relationship between renal function and UC incidence were calculated using Cox proportional hazards models with a time-on-study scale. Since the MJ database provides only monthly information for dates, we assumed that every baseline started on the 15th of that month and year to obtain more precise estimates of survival, as recommended in the literature [18]. The same approach was used if participants had partial dates for UC diagnosis or death. Three models were developed and reported: model 1, a crude model with no adjustment; model 2, adjusted for age and gender; and model 3 (main model presented), further adjusted for education, smoking status, long-term medication, proteinuria, hematuria, DM, and PM_2.5_ exposure.

### 2.4 Sensitivity analysis

To explore the possibility of reverse causality, we split the follow-up duration into two periods (≤10 yr and >10 yr) and estimated separate HRs for each interval as a sensitivity analysis. We also constructed another competing-risk model considering death before UC incidence as a competing event using the Fine and Gray [19] approach. An additional sensitivity analysis was performed by including only participants who provided residential coordinates in the questionnaire.

## 3 Results

### 3.1 Baseline characteristics

During a median follow-up of 10.3 yr, 383 cases of UC occurred (Table 1). The median age at UC diagnosis was 70.0 yr. The age-standardized incidence rate for UC was 8.87 per 10^5^ population overall, and 12.32 and 5.53 for men and women, respectively. The mean age was 43.2 yr. Compared to individuals with normal renal function, participants with low eGFR tended to be older and have higher body mass index, lower education level, and higher comorbidity (all *p* <  0.0001). Individuals with normal renal function were more likely to have healthy lifestyle behaviors and less likely to be taking long-term medications than the other eGFR groups (all *p* <  0.0001).

**Table 1:** Baseline characteristics by eGFR strata

Variable	Overall	eGFR (ml/min/1.73 m^2^)				*p* value^a^
		≥90	60–89	45–59	<45	
Patients (*n*)	372 008	165 605	191 525	12 135	2743	
Urothelial carcinoma, *n* (%)^d^	383 (0.10)	62 (0.04)	227 (0.12)	73 (0.60)	21 (0.77)	<0.0001
Median follow-up, yr (IQR)	10.3 (7.7)	10.5 (7.4)	10.0 (7.8)	10.8 (7.4)	9.0 (7.7)	<0.0001
Mean age, yr (SD)	43.2 (11.9)	38.3 (3.4)	45.8 (12.1)	63.4 (10.7)	65.9 (11.9)	<0.0001
Male, *n* (%)	182 068 (49)	65 058 (39)	108 404 (57)	7121 (59)	1485 (54)	<0.0001
High education level, *n* (%)^b^	205 385 (58)	103 134 (65)	99 242 (54)	2597 (23)	412 (16)	<0.0001
Smoking status, *n* (%)^b^						
Never	255 538 (73)	119 391 (76)	126 494 (70)	7836 (69)	1817 (71)	<0.0001
Former	22 552 (6)	7568 (5)	13 332 (7)	1330 (12)	322 (13)	
Current	74 178 (21)	30 074 (19)	41 446 (23)	2233 (20)	425 (17)	
Drinking habits, *n* (%)^b^						
Never	278 113 (81)	128 980 (84)	138 981 (79)	8240 (76)	1912 (78)	<0.0001
Former	10 385 (3)	3488 (2)	5844 (3)	788 (7)	265 (11)	
Current	53 205 (16)	20 409 (13)	30 711 (17)	1807 (17)	278 (11)	
Long-term medication, *n* (%)^b^	106 204 (29)	36 174 (10)	60 271 (37)	7587 (63)	2172 (79)	<0.0001
Body mass index, *n* (%)^b^						
<18.5 kg/m^2^	23 862 (6)	14 785 (9)	8656 (5)	321 (3)	100 (4)	<0.0001
18.5–24 kg/m^2^	199 717 (54)	97 349 (59)	96 283 (50)	4925 (41)	1160 (42)	
24–27 kg/m^2^	93 279 (25)	33 145 (20)	55 041 (29)	4264 (35)	829 (30)	
27 kg/m^2^	55 012 (15)	20 257 (12)	31 487 (16)	2618 (22)	650 (24)	
Proteinuria, *n* (%)^b^	18 998 (5)	5950 (4)	10 034 (5)	1723 (14)	1291 (48)	<0.0001
Hematuria, *n* (%)^b^	90 488 (25)	38 288 (24)	46 917 (25)	3979 (33)	1304 (49)	<0.0001
Medical history, *n* (%)						
Hypertension^b^	34 809 (9)	7157 (4)	21 405 (11)	4695 (39)	1552 (57)	<0.0001
Cardiovascular disease^b^	13 586 (4)	3152 (2)	7853 (4)	1918 (16)	663 (24)	<0.0001
Diabetes mellitus^b^	21 926 (6)	6570 (4)	12 469 (7)	2074 (17)	813 (30)	<0.0001
Nephrosis^b^	5332 (1)	1926 (1)	2457 (1)	424 (3)	525 (19)	<0.0001
Metabolic syndrome	49 221 (13)	14 427 (9)	29 265 (15)	4208 (35)	1321 (48)	<0.0001
Mean 2-yr PM_2.5_, *n* (%)^b^						
<18.25 μg/m^3^	92 587 (25)	37 722 (23)	50 163 (26)	3768 (31)	934 (34)	<0.0001
18.25–22.35 μg/m^3^	93 695 (25)	39 682 (24)	49 719 (26)	3531 (29)	763 (28)	
22.35–25.1 μg/m^3^	92 105 (25)	45 857 (28)	43 576 (23)	2205 (18)	467 (17)	
≥25.1 μg/m^3^	93 265 (25)	42 186 (25)	47 888 (25)	2618 (22)	573 (21)	
Live in HACA, *n* (%) b, c	239 (0.06)	80 (0.05)	135 (0.07)	16 (0.13)	8 (0.29)	<0.0001
Residential address, *n* (%)	308 514 (83)	132 759 (80)	161 490 (84)	11 622 (96)	2643 (96)	<0.0001
Mean eGFR, ml/min/1.73 m^2^ (SD)	87.6 (5.8)	102.0 (8.9)	78.0 (7.6)	54.2 (4.0)	32.8 (11.3)	<0.0001

eGFR = estimated glomerular filtration rate; HACA = high arsenic contamination area; IQR = interquartile range; PM, particulate matter; SD = standard deviation.

a The *p* values are based on the Kruskal-Wallis test for follow-up year, analysis of variance for age and eGFR, or Pearson’s χ^2^ test for gender, education, smoking status, alcohol consumption, long-term medication, body mass index, proteinuria, hematuria, proportions of comorbidities, and mean 2-year PM_2.5_ concentration. All statistical tests were two-sided.

b This variable had missing data.

c HACAs are Budai and Yizhu townships in Chiayi County, and Syuejia and Beimen districts in Tainan City.

d List of histology codes: 8000, 8010, 8120, 8130, 8246.

### 3.2 Association between renal function and UC incidence

**Fig. 2:**
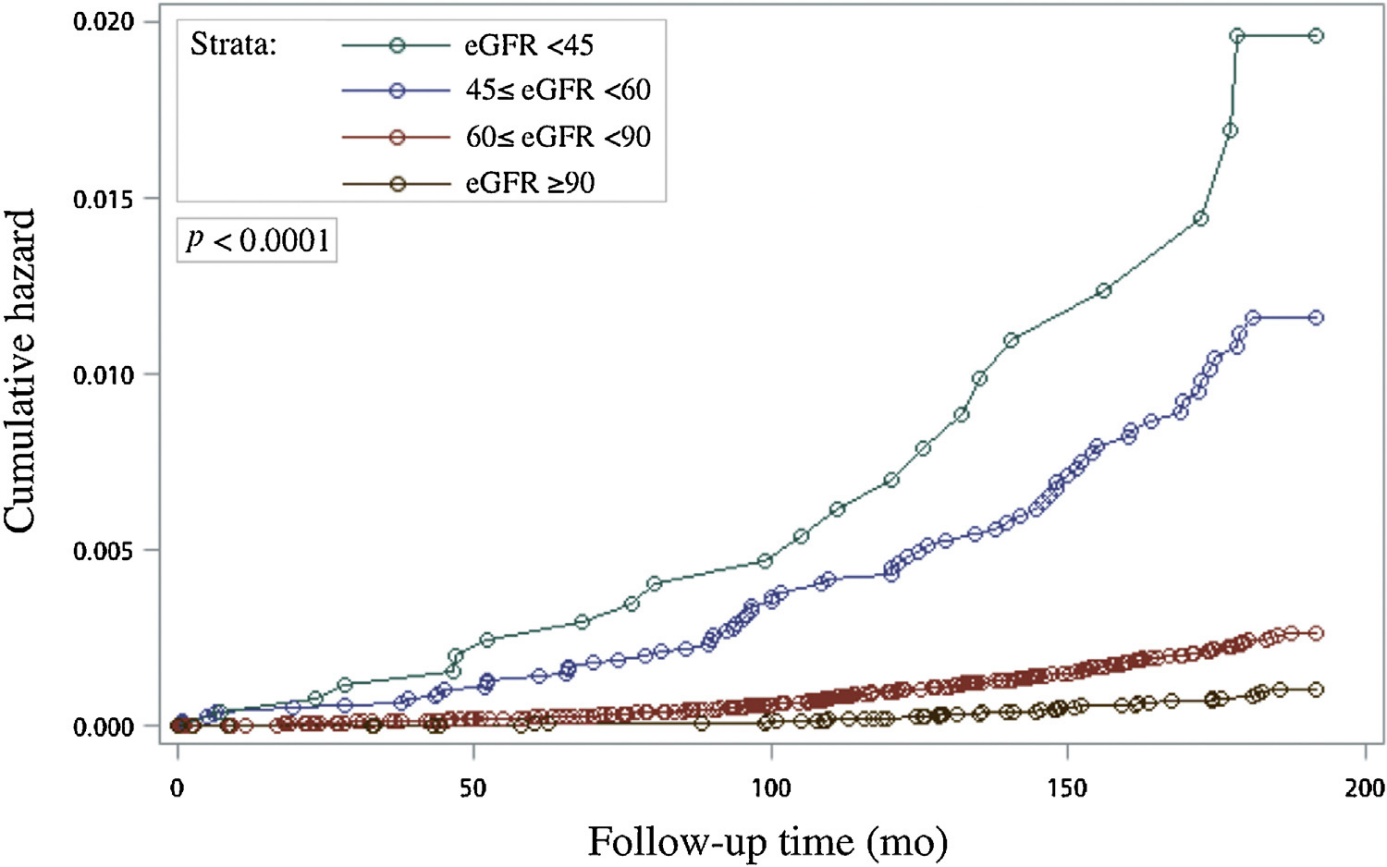
Nelson-Aalen estimates of the cumulative UC hazard by stratified estimated glomerular filtration rate (eGFR in ml/min/1.73 m^2^).

Cumulative hazard curves for UC are shown in Figure 2. Significant differences were noted across the four eGFR strata (*p* <  0.0001). Table 2 shows the association between renal function and UC incidence. After adjusting for age, gender, education, smoking status, long-term medication, proteinuria, hematuria, DM, and long-term PM_2.5_ exposure (model 3), a significant dose-response relationship between renal function and UC incidence was noted (*p*_trend_ < 0.01). Compared with eGFR ≥90 ml/min/1.73 m^2^, lower eGFR ranges were associated with significantly higher UC risk: HR 1.36 (95% CI 0.98–1.88) for eGFR of 60–89 ml/min/1.73 m^2^, HR 1.86 (95% CI 1.22–2.84) for eGFR of 45–59 ml/min/1.73 m^2^, and HR 1.95 (95% CI 1.06–3.56) for eGFR of <45 ml/min/1.73 m^2^. Furthermore, smoking (HR 1.34, 95% CI 1.02–1.77) and long-term exposure to PM_2.5_ ≥25.1 μg/m^3^ (HR 1.54, 95% CI 1.14–2.09) were identified as significant risk factors for UC. A significant dose-response relationship between PM_2.5_ concentration and UC risk was also noted (*p*_trend_ < 0.01).

**Table 2:** Association between eGFR and UC incidence^a^

Variable	Model 1 (*n* = 372 008, UC = 383)			Model 2 (*n* = 372 008, UC = 383)			Model 3 (*n* = 333 450, UC = 344)^b^		
	Crude HR (95% CI)	*p* value	*p*_trend_	AHR (95% CI)	*p* value	*p*_trend_	AHR (95% CI)	*p* value	*p*_trend_
eGFR									
≥90 ml/min/1.73 m^2^	Reference		<0.0001	Reference		0.0001	Reference		0.004
60–89 ml/min/1.73 m^2^	3.11 (2.35–4.12)	***		1.23 (0.91–1.66)			1.36 (0.98–1.88)		
45–59 ml/min/1.73 m^2^	14.49 (10.32–20.33)	***		1.82 (1.22–2.70)	**		1.86 (1.22–2.84)	**	
<45 ml/min/1.73 m^2^	23.26 (14.18–38.15)	***		2.47 (1.43–4.26)	**		1.95 (1.06–3.56)	*	
Age	–			1.09 (1.08–1.10)	***		1.08 (1.07–1.09)	***	
Male	–			2.04 (1.65–2.53)	***		1.95 (1.49–2.57)	***	
High education level	–			–			0.92 (0.71–1.20)		
Smoking status									
Former	–						1.32 (0.94–1.86)		0.030
Current							1.34 (1.02–1.77)	*	
Long-term medication	–			–			1.10 (0.87–1.39)		
Proteinuria	–			–			1.67 (1.22–2.27)	**	
Hematuria	–			–			1.27 (1.01–1.60)	*	
Diabetes mellitus	–			–			1.81 (1.37–2.38)	***	
Mean 2-yr PM_2.5_									
<18.25 μg/m^3^	–			–			Reference		0.009
18.25–22.35 μg/m^3^							0.82 (0.62–1.07)		
22.35–25.1 μg/m^3^							1.05 (0.76–1.45)		
≥25.1 μg/m^3^							1.54 (1.14–2.09)	**	

AHR = adjusted HR; CI = confidence interval; eGFR = estimated glomerular filtration rate; HR = hazard ratio; PM = particulate matter; UC = urothelial carcinoma.

a The effect size of each variable was adjusted for the other variables in each model.

b Analyses were restricted to participants for whom complete information for all covariates in the model was available.

* *p* <  0.05; ** *p* <  0.01; *** *p* <  0.001.

### 3.3 Sensitivity analysis

To explore the possibility of reverse causality, we performed a sensitivity analysis in which separate HRs were estimated for follow-up durations of ≤10 yr and >10 yr (Table 3). The HR remained elevated beyond 10 yr from enrollment, suggesting that reverse causality explains a limited part of the relationship and strengthening the notion that lower eGFR may be a risk factor for UC. The other two sensitivity analyses are shown in Supplementary Table 2: one treated death before cancer incidence as a competing event using the subdistribution hazard approach; the other model was constructed after excluding participants who provided coordinates for their company and not their residential location. The results from both analyses were not considerably different from those for the main model.

**Table 3:** Association between eGFR and UC incidence by follow-up duration a

eGFR	Follow-up ≤10 yr (*n* = 157 880, UC = 189)			Follow-up >10 yr (*n* = 175 570, UC = 155)		
	AHR (95% CI)	*p* value	*p*_trend_	AHR (95% CI)	*p* value	*p*_trend_
≥90 ml/min/1.73 m^2^	Reference		0.154	Reference		0.043
60–89 ml/min/1.73 m^2^	1.87 (1.14–3.05)	*		0.92 (0.63–1.53)		
45–59 ml/min/1.73 m^2^	2.10 (1.13–3.94)	*		1.64 (0.90–2.97)		
<45 ml/min/1.73 m^2^	1.54 (0.66–3.61)			2.12 (0.86–5.20)		

AHR = adjusted hazard ratio; CI = confidence interval; eGFR = estimated glomerular filtration rate; PM = particulate matter; UC = urothelial carcinoma.

a Adjusted for age, gender, education, smoking status, long-term medication, proteinuria, hematuria, diabetes mellitus, and long-term PM_2.5_ exposure (model 3). Analyses were restricted to participants for whom complete data for all covariates in the model was available.

* *p* <  0.05.

### 3.4 Stratified analysis

Given the knowledge that men have higher risks for both lower renal function and UC incidence, we conducted a gender-stratified analysis (Table 4). A stronger dose-response relationship between eGFR and UC was found for women (HR 1.22, 95% CI 0.74–2.02; HR 1.49, 95% CI 0.71–3.13; and HR 2.97, 95% CI 1.19–7.40 for eGFR of 60–89, 45–59, and <45 ml/min/1.73 m^2^, respectively) than for men. Gender differences in the effect of smoking on UC were also found. Smoking was a significant risk factor for UC for men (HR 1.40, 95% CI 1.05–1.88) but not for women (HR 1.00, 95% CI 0.37–2.73).

**Table 4:** Gender-stratified analysis of the association between eGFR and UC incidence^a^.

Variable	Male (*n* = 170 226, UC = 233)			Female (*n* = 163 224, UC = 111)		
	AHR (95% CI)	*p* value	*p*_trend_	AHR (95% CI)	*p* value	*p*_trend_
eGFR						
≥90 ml/min/1.73 m^2^	Reference		0.032	Reference		0.041
60–89 ml/min/1.73 m^2^	1.47 (0.95–2.26)			1.22 (0.74–2.02)		
45–59 ml/min/1.73 m^2^	2.09 (1.22–3.56)	**		1.49 (0.71–3.13)		
<45 ml/min/1.73 m^2^	1.52 (0.67–3.44)			2.97 (1.19–7.40)	*	
Age	1.08 (1.06–1.09)	***		1.09 (1.07–1.11)	***	
High education level	0.97 (0.73–1.30)			0.77 (0.42–1.40)		
Smoking status						
Former^b^	1.41 (0.99–2.01)		0.017	0.00 (0.00–NA)		0.727
Current	1.40 (1.05–1.88)	*		1.00 (0.37–2.73)		
Long-term medication	1.30 (0.98–1.73)			0.79 (0.52–1.18)		
Proteinuria	1.48 (1.02–2.16)	*		2.07 (1.20–3.59)	**	
Hematuria	1.49 (1.12–1.98)	**		0.96 (0.65–1.41)		
Diabetes mellitus	1.96 (1.42–2.72)	***		1.45 (0.86–2.44)		
Mean 2-yr PM_2.5_						
<18.25 μg/m^3^	Reference		0.034	Reference		0.124
18.25–22.35 μg/m^3^	0.77 (0.55–1.07)			0.92 (0.57–1.47)		
22.35–25.1 μg/m^3^	1.05 (0.71–1.56)			1.02 (0.56–1.84)		
≥25.1 μg/m^3^	1.51 (1.05–2.19)	*		1.60 (0.93–2.75)		

AHR = adjusted hazard ratio; CI = confidence interval; eGFR = estimated glomerular filtration rate; PM = particulate matter; UC = urothelial carcinoma.

a Adjusted for age, education, smoking status, long-term medication, proteinuria, hematuria, diabetes mellitus, and long-term PM_2.5_ exposure (model 3). Analyses were restricted to participants for whom complete data for all covariates in the model was available.

b There were no former smokers among females.

* *p* <  0.05; ** *p* <  0.01; *** *p* <  0.001.

Taiwan uniquely has a higher proportion of UTUCs among all UCs in comparison to Western countries, and the proportion of women with UTUC is surprisingly higher than for men [20]. Supplementary Table 3 shows the proportions of UC events for upper and lower urinary tract sites by gender in this study. Owing to the unique incidence pattern for Taiwan, we performed an analysis stratified by cancer site (Supplementary Table 4). A dose-response relationship between eGFR and UC was observed for both UTUC and LTUC. However, male gender was not a significant risk factor for UTUC (HR 1.16, 95% CI 0.52–2.59). In addition, smoking was only a risk factor for LTUC (HR 1.36, 95% CI 1.02–1.81) and the risk associated with PM_2.5_ was much greater for UTUC (HR 4.36, 95% CI 1.38–13.77; HR 8.80, 95% CI 2.84–27.23 for PM_2.5_ of 22.35–25.09 and >25.09 μg/m^3^, respectively) than for LTUC.

## 4 Discussion

In this retrospective cohort study, we identified a strong and significant dose-response relationship between low renal function and UC incidence. Besides the well-established risk factor of smoking, we also identified PM_2.5_ exposure as a possible risk factor for UC.

### 4.1 Comparison with other studies

Two previous studies [4], [6] reported a U-shaped relationship between eGFR and overall cancer incidence: individuals with eGFR ≥105 ml/min/1.73 m^2^ had slightly higher risk than those with eGFR of 60–89 ml/min/1.73 m^2^. By contrast, after further classifying eGFR into five categories (≥110, 90–109, 60–89, 45–59, and <45 ml/min/1.73 m^2^) we did not observe a U-shaped relationship in our study (Supplementary Table 5).

Mechanisms that may contribute to tumorigenesis include accumulation of carcinogenic compounds, a weakened immune system, systemic inflammation, and impaired DNA repair mechanisms [7], [8], [9]. Some other possible mechanisms that have been discussed are alterations of the renin-angiotensin system [21] and some medications used by CKD patients [1], [8]. All the aforementioned mechanisms may result in an increase in the risk of cancer.

Smoking is the most well-established risk factor for bladder cancer, and smoking cessation may help in decreasing bladder cancer risk [22]. We also found that individuals who lived in areas with higher PM_2.5_ concentrations were more likely to develop UC (*p*_trend_ < 0.01), consistent with previous studies [12], [13]. The most common hypothesis for the mechanism underlying this association is that PM_2.5_ exposure may lead to systemic inflammation and oxidative stress [23]. In addition, PM_2.5_ particles can enter the circulatory system through the alveoli and have adverse effects on remote organs [23]. The toxicity of chemical compounds such as heavy metals and polycyclic aromatic hydrocarbons that adhere to PM may also pose risks [24]. However, a meta-analysis indicated that PM_2.5_ exposure has no association with UC [25]. It is possible that because of lower PM concentrations in Western countries where the studies were carried out, they did not detect this link. Thus, more studies on different ambient PM_2.5_ concentrations are warranted to validate this relationship.

### 4.2 Strengths and limitations

This study has several strengths. First, it is a large, population-based cohort study with favorable generalizability. This is confirmed by the fact that our findings for UC incidence (data shown in Supplementary Table 6) are similar to those in the *2017 Taiwan Cancer Registry Annual Report* (https://www.hpa.gov.tw/Pages/Detail.aspx?nodeid=269&pid=12235), which is 10.15 per 10^5^ population overall, and 12.99 and 7.66 for men and women, respectively. Second, the study had longer follow-up than similar studies, and we conducted a sensitivity analysis to explore the possibility of reverse causality.

There are also some limitations. Patients with ESRD have different characteristics from others and have a higher cancer risk [3]. Owing to the lack of information on medical history, we were not able to exclude patients with ESRD before the analysis. Checking of eGFR data revealed that there were only 305 individuals with eGFR <15 ml/min/1.73 m^2^ at baseline among the entire study population, of whom five developed UC before the end of the follow-up period. In addition, as our study population was from a database for routine health examinations, we presume that only a few people may have had ESRD. Another limitation is the lack of detailed information about the clinical condition of patients at the time of their UC diagnosis. It would be better if we could take into consideration complications caused by UC that could also impair renal function and adjust for these potential confounders. However, we found that very few people (0.01%) reported ever having had nephrosis before baseline. Combined with the view that UC has a relatively short lead-time and the long follow-up duration for the UC groups (9.4 yr; Supplementary Table 8), we believe that it is unlikely that complications caused by UC could affect renal function in our cohort. Moreover, it is theoretically plausible that patients with poorer renal function have a more intense follow-up schedule and may have ended up with a higher UC detection rate compared to healthy patients. The data and biological samples from the MJ Health Database were voluntarily donated by participants, who were apparently healthy and receiving physical examination services from the MJ Health Management Institution. The number of donations, as well as the time interval between each donation, varies from one participant to another. However, from the similar rates for UC incidence (Supplementary Table 6) and tumor stage (Supplementary Table 7) we observed in the MJ database compared to Taiwan, we would optimistically speculate that the disease risk for participants in the MJ cohort is close to that of the general population in Taiwan. In addition, previous evidence suggests that even short-term exposure or exposure in recent years can serve as a favorable surrogate for long-term PM exposure because of the high correlation in annual exposure between different geographical locations over time [26]. Thus, we conducted a sensitivity analysis restricted to participants who gave their residential address, and the results were similar to the original results.

## 5 Conclusions

In conclusion, lower renal function is an independent risk factor for UC incidence. Even a mild reduction in renal function leads to higher UC risk, indicating that improving the management of CKD is critical in the prevention of UC. This finding may also aid in the development of a complete cancer screening strategy: individuals with lower renal function should monitor their health not only in terms of the kidneys themselves but also the other organs in the urinary system. We also found that smoking and long-term exposure to PM_2.5_ are both risk factors for UC. Tobacco and air pollution control policies should be strictly implemented for prevention of UC and many other adverse health outcomes.

  ***Author contributions***: Ta-Chien Chan had full access to all the data in the study and takes responsibility for the integrity of the data and the accuracy of the data analysis.

  *Study concept and design*: Chan, Chuang, I-F. Lin.

*Acquisition of data*: Chan, Lao, C. Lin.

*Analysis and interpretation of data*: Chuang, Chan, I-F. Lin.

*Drafting of the manuscript*: Chuang, Chan.

*Critical revision of the manuscript for important intellectual content*: Chan.

*Statistical analysis*: Chuang, I-F. Lin.

*Obtaining funding*: Chan.

*Administrative, technical, or material support*: Chan.

*Supervision*: Chan, I-F. Lin.

*Other*: None.

  ***Financial disclosures:*** Ta-Chien Chan certifies that all conflicts of interest, including specific financial interests and relationships and affiliations relevant to the subject matter or materials discussed in the manuscript (eg, employment/affiliation, grants or funding, consultancies, honoraria, stock ownership or options, expert testimony, royalties, or patents filed, received, or pending), are the following: None.

  ***Funding/Support and role of the sponsor*:** This research was supported by a grant from the Ministry of Science and Technology, Taiwan (MOST- 108-2628-M-001-008-MY3) and a Multidisciplinary Health Cloud Research Program: Technology Development and Application of Big Health Data grant from Academia Sinica. The funders had no role in study design, data collection and analysis, decision to publish, or preparation of the manuscript.

  ***Ethics approval and consent to participate*:** Informed consent was obtained to authorize data processing and analysis. Ethical reviews were approved by the institutional review board of Biomedical Science Research, Academia Sinica (AS-IRB-BM-17044). Individual-identifying data were removed and patients remained anonymous during the entire study.

  ***Data sharing statement*:** The data that support the findings of this study are available from the MJ Health Research Foundation and Ministry of Health and Welfare, Taiwan, but restrictions apply to the availability of these data, which were under approval for the current study and so are not publicly available. The linked data set used in this study had to be analyzed in person in the Health and Welfare Data Science Center, Ministry of Health and Welfare, Taiwan.

  ***Acknowledgments*:** We thank Dr. Chung-You Tsai from Far Eastern Memorial Hospital, Taiwan and Dr. Hugo Y.‑H. Lin from Kaohsiung Municipal Ta-Tung Hospital, Taiwan for providing clinical suggestions for the study. We would also like to thank the MJ Health Research Foundation for authorizing use of the MJ health data (authorization code: MJHRF2017009A). Our interpretations and conclusions do not necessarily represent the views of the MJ Health Research Foundation.
