# Supplementary material for: The Association Between Renal Function Decline and the Incidence of Urothelial Carcinoma: A 16-year Retrospective Cohort Study in Taiwan
Source: Eur Urol Open Sci. 2021 Mar 11;27:1–9. doi: 10.1016/j.euros.2021.02.004 (PMC8317881; doi:10.1016/j.euros.2021.02.004)
Supplement: Supplementary file 1 [file mmc1.docx]

**Supplementary Tables**

**Supplementary Table 1.** Histology Codes (ICD-O-3 M-code) Captured from the UC Cases

| **Histology Code** | **Term** |
| --- | --- |
| 8000 | Neoplasm, malignant  Blastoma, NOS  Tumor, malignant, NOS  Cancer  Malignancy  Unclassified tumor, malignant |
| 8010 | Intraepithelial carcinoma, NOS |
|  | Carcinoma, NOS  Epithelial tumor, malignant  Heterotopia-associated carcinoma |
| 8120 | Transitional cell carcinoma in situ  Urothelial carcinoma in situ |
|  | Transitional cell carcinoma, NOS  Transitional carcinoma  Urothelial carcinoma, NOS |
| 8130 | Papillary transitional cell carcinoma, non-invasive  Papillary urothelial carcinoma, non-invasive |
|  | Papillary transitional cell carcinoma  Papillary urothelial carcinoma |
| 8246 | Neuroendocrine carcinoma, NOS  Poorly differentiated neuroendocrine neoplasm |

**Supplementary Table 2.** Association Between eGFR and UC Incidence in Sensitivity Analysis

|  | **Sensitivity Analysis^a^** | | | | | **Sensitivity Analysis^b^** | | | | |
| --- | --- | --- | --- | --- | --- | --- | --- | --- | --- | --- |
|  | N=333,450 (UC=344) | | | | | N=277,818 (UC=317) | | | | |
| **Variable** | **Adjusted HR (95%CI)** | | | ***P*** | ***P_trend_*** | **Adjusted HR (95%CI)** | | | ***P*** | ***P_trend_*** |
| **eGFR strata** |  |  | |  |  |  |  | |  |  |
| eGFR≥90 | **ref** | | |  | 0.029 | **ref** | | |  | 0.004 |
| 60≤eGFR<90 | **1.43** | 1.03, | 2.00 | * |  | **1.25** | 0.89, | 1.76 |  |  |
| 45≤eGFR<60 | **1.88** | 1.19, | 2.96 | ** |  | **1.82** | 1.18, | 2.81 | ** |  |
| eGFR<45 | **1.52** | 0.73, | 2.91 |  |  | **1.93** | 1.04, | 3.56 | * |  |
| **Age** | **1.07** | 1.06, | 1.08 | *** |  | **1.08** | 1.07, | 1.09 | *** |  |
| **Male** | **1.89** | 1.45, | 2.48 | *** |  | **1.90** | 1.44, | 2.53 | *** |  |
| **High education level** | **0.90** | 0.69, | 1.19 |  |  | **0.95** | 0.72, | 1.25 |  |  |
| **Smoking status** |  |  | |  |  |  |  | |  |  |
| Former | **1.27** | 0.91, | 1.77 |  | 0.070 | **1.34** | 0.94, | 1.92 |  | 0.033 |
| Current | **1.27** | 0.97, | 1.67 |  |  | **1.35** | 1.01, | 1.81 | * |  |
| **Long-term medication** | **1.09** | 0.85, | 1.38 |  |  | **1.08** | 0.85, | 1.38 |  |  |
| **Proteinuria** | **1.50** | 1.09, | 2.04 | * |  | **1.71** | 1.24, | 2.35 | *** |  |
| **Hematuria** | **1.27** | 1.01, | 1.61 | * |  | **1.26** | 0.99, | 1.60 |  |  |
| **Diabetes Mellitus** | **1.72** | 1.29, | 2.29 | *** |  | **1.71** | 1.28, | 2.29 | *** |  |
| **Mean 2-year PM_2.5_** |  |  | |  |  |  |  | |  |  |
| PM_2.5_<18.25 | **ref** | | |  | 0.051 | **ref** | | |  | 0.013 |
| 18.25≤PM_2.5_<22.35 | **0.83** | 0.64, | 1.10 |  |  | **0.87** | 0.66, | 1.14 |  |  |
| 22.35≤PM_2.5_<25.1 | **1.00** | 0.73, | 1.38 |  |  | **1.13** | 0.80, | 1.58 |  |  |
| PM_2.5_≥25.1 | **1.39** | 1.03, | 1.86 | * |  | **1.52** | 1.11, | 2.10 | * |  |

**Supplementary Table 3.** Counts of Incident UCs by Gender and Site

| **Cancer Site** | **Overall** | | **Male** | | **Female** | |
| --- | --- | --- | --- | --- | --- | --- |
| **UTUC** | 36 | (9.4%) | 18 | (7.1%) | 18 | (13.8%) |
| **LTUC** | 347 | (90.6%) | 235 | (92.9%) | 112 | (86.2%) |
| **Total (UC)** | 383 | | 253 | | 130 | |
| Abbreviations: LTUC, lower tract urothelial carcinoma; UC, urothelial carcinoma; UTUC, upper tract urothelial carcinoma. | | | | | | |

**Supplementary Table 4.** Cancer Site-stratified Analysis of the Association Between eGFR and UC Incidence

|  |  | **UTUC** (N=333,450, UC=36) | | | | |  | **LTUC** (N=333,450, UC=308) | | | | |
| --- | --- | --- | --- | --- | --- | --- | --- | --- | --- | --- | --- | --- |
| **Variable** |  | **Adjusted HR (95%CI)** | | | ***P*** | ***P_trend_*** |  | **Adjusted HR (95%CI)** | | | ***P*** | ***P_trend_*** |
| **eGFR strata** |  |  |  | |  |  |  |  |  | |  |  |
| eGFR≥90 |  | **ref** | | |  | 0.043 |  | **ref** | | |  | 0.017 |
| 60≤eGFR<90 |  | **2.04** | 0.67, | 6.25 |  |  |  | **1.30** | 0.93, | 1.83 |  |  |
| 45≤eGFR<60 |  | **2.00** | 0.46, | 8.70 |  |  |  | **1.84** | 1.18, | 2.86 | ** |  |
| eGFR<45 |  | **7.35** | 1.49, | 36.34 | * |  |  | **1.58** | 0.81, | 3.09 |  |  |
| **Age** |  | **1.09** | 1.05, | 1.12 | *** |  |  | **1.08** | 1.07, | 1.09 | *** |  |
| **Male** |  | **1.16** | 0.52, | 2.59 |  |  |  | **2.10** | 1.57, | 2.81 | *** |  |
| **High education level** |  | **0.95** | 0.41, | 2.20 |  |  |  | **0.92** | 0.70, | 1.22 |  |  |
| **Smoking status** |  |  |  | |  |  |  |  |  | |  |  |
| Former |  | **0.92** | 0.26, | 3.29 |  | 0.775 |  | **1.36** | 0.95, | 1.95 |  | 0.030 |
| Current |  | **1.16** | 0.45, | 2.99 |  |  |  | **1.36** | 1.02, | 1.81 | * |  |
| **Long-term medication** |  | **1.42** | 0.68, | 2.97 |  |  |  | **1.07** | 0.83, | 1.37 |  |  |
| **Proteinuria** |  | **0.57** | 1.16, | 2.01 |  |  |  | **1.84** | 1.34, | 2.53 | *** |  |
| **Hematuria** |  | **2.20** | 1.11, | 4.35 | * |  |  | **1.19** | 0.93, | 1.52 |  |  |
| **Diabetes mellitus** |  | **2.01** | 0.87, | 4.66 |  |  |  | **1.79** | 1.33, | 2.40 | *** |  |
| **Mean 2-year PM_2.5_** |  |  |  | |  |  |  |  |  | |  |  |
| PM_2.5_<18.25 |  | **ref** | | | <0.0001 | |  | **ref** | | |  | 0.185 |
| 18.25≤PM_2.5_<22.35 |  | **1.58** | 0.54, | 4.64 |  |  |  | **0.78** | 0.59, | 1.03 |  |  |
| 22.35≤PM_2.5_<25.1 |  | **4.36** | 1.38, | 13.77 | * |  |  | **0.92** | 0.65, | 1.30 |  |  |
| PM_2.5_≥25.1 |  | **8.80** | 2.84, | 27.23 | *** |  |  | **1.30** | 0.94, | 1.80 |  |  |
| Note: **P* < .05; ***P* < .01; ****P* < .001.  Adjusted for age, gender, education, smoking status, long-term medication, proteinuria, hematuria, diabetes mellitus, and long-term PM_2.5_ exposure (Model 3). Analyses were restricted to participants who had complete information for all covariates in the model.  Abbreviations: CI, confidence interval; eGFR, estimated glomerular filtration rate; HR, hazard ratio; LTUC, lower tract urothelial carcinoma; PM, particulate matter; UC, urothelial carcinoma; UTUC, upper tract urothelial carcinoma | | | | | | | | | | | | |

**Supplementary Table 5.** Association Between eGFR and UC Incidence: classified eGFR into 5 categories

|  |  | **Model 3**^a^ (N=333,450, UC=344) | | | | |
| --- | --- | --- | --- | --- | --- | --- |
| **Variable** |  | **Adjusted HR (95%CI)** | | | ***P*** | ***P_trend_*** |
| **eGFR strata** |  |  |  | |  |  |
| eGFR≥110 |  | **ref** | | |  | 0.002 |
| 90≤eGFR<110 |  | **1.63** | 0.58, | 4.56 |  |  |
| 60≤eGFR<90 |  | **2.14** | 0.77, | 5.90 |  |  |
| 45≤eGFR<60 |  | **2.94** | 1.02, | 8.51 | * |  |
| eGFR<45 |  | **3.08** | 0.98, | 9.73 |  |  |
| Note: * *P* < .05; ** *P* < .01; ****P* < .001.  Adjusted for age, gender, education, smoking status, long-term medication, proteinuria, hematuria, diabetes mellitus, and long-term PM_2.5_ exposure (Model 3). Analyses were restricted to participants who had complete information for all covariates in the model.  ^a^ Analyses were restricted to participants who had complete information for all covariates in the model.  Abbreviations: CI, confidence interval; eGFR, estimated glomerular filtration rate; HR, hazard ratio; PM, particulate matter; UC, urothelial carcinoma. | | | | | | |

**Supplementary Table 6.** Age-standardized Incidence Rate (ASR) by Primary Anatomic Site and Sex [1]

| **ICD-O-3** | **Primary Anatomic Sites** | **Total** | **Male** | **Female** |
| --- | --- | --- | --- | --- |
| C65 | Renal Pelvis | 2.39 | 2.21 | 2.54 |
| C66 | Ureter | 1.70 | 1.53 | 1.84 |
| C67 | Bladder | 5.97 | 9.11 | 3.23 |
| C68.0-C68.1 | Urethra and Paraurethral Gland | 0.09 | 0.14 | 0.05 |
| **Total** |  | **10.15** | **12.99** | **7.66** |
| Note: ASR was calculated using the 2000 standard population data from the World Health Organization. (Unit: per 10^5^ person-years.)  The ASR of UC was not directly presented in the 2017 Taiwan Cancer Registry Annual Report. We calculate an approximation by combining incidence of cancer sites coded: C65.0-C68.1, without specifying histology. | | | | |

**Supplementary Table 7.** Tumor Clinical Stage of Incident UC in MJ and 2017 Taiwan Cancer Registry Annual Report [1]

| **Tumor Stage** | **MJ** | | **2017 Taiwan Cancer**  **Registry Annual Report** | |
| --- | --- | --- | --- | --- |
| Stage 0 | 105 | (27.42%) | 946 | (28.64%) |
| Stage 1 | 116 | (30.29%) | 1173 | (35.51%) |
| Stage 2 | 38 | (9.92%) | 564 | (17.08%) |
| Stage 3 | 17 | (4,44%) | 245 | (7.42%) |
| Stage 4 | 26 | (6.79%) | 245 | (7.42%) |
| Unspecified | 81 | (21.15%) | 130 | (3.94%) |
| **Total** | **383** | | **3303** | |
| Note: The number retrieved from 2017 Taiwan Cancer Registry Annual Report included bladder cancer only, coded: C67.0-C67.9. | | | | |

**Supplementary Table 8.** Distribution of Follow-Up Duration (years), Stratified by UC Incident

|  | **Overall** | **Non-UC** | **UC** |
| --- | --- | --- | --- |
|  | N=372,008 | N=371,625 | N=383 |
| **Median±IQR** | 10.29±7.67 | 10.29±7.67 | 9.38±6.04 |
| **Mean±SD** | 9.87±4.40 | 9.87±4.40 | 9.02±4.01 |
| **(Q1, Q3)** | (6.29, 13.96) | (6.29, 13.96) | (6.28, 12.32) |
| **(Min, Max)** | (0.02, 15.96) | (0.02, 15.96) | (0.03, 15.61) |
| Abbreviations: UC, urothelial carcinoma; IQR, interquartile range; SD, standard deviation; Q1, first quartile; Q3, third quartile; Min, minimum; Max, maximum. | | | |

**References**

[1] Taiwan Health Promotion Administration Ministry of Health and Welfare. 2017 Taiwan Cancer Registry Annual Report. 2019.
